# ZmLBD2 a maize (*Zea mays* L.) lateral organ boundaries domain (LBD) transcription factor enhances drought tolerance in transgenic *Arabidopsis thaliana*

**DOI:** 10.3389/fpls.2022.1000149

**Published:** 2022-10-13

**Authors:** Peng Jiao, Xiaotong Wei, Zhenzhong Jiang, Siyan Liu, Shuyan Guan, Yiyong Ma

**Affiliations:** ^1^ College of Life Sciences, Jilin Agricultural University, Changchun, China; ^2^ Joint International Research Laboratory of Modern Agricultural Technology, Ministry of Education, Changchun, China; ^3^ College of Agronomy, Jilin Agricultural University, Changchun, China

**Keywords:** lateral organ boundaries domain, LBD2, transcription factor, drought tolerance, maize

## Abstract

Maize (*Zea mays* L.) is an annual gramineous herb and is among the world’s most important crop species. Drought is the main factor contributing to maize yield reduction. The lateral organ boundaries domain (LBD) proteins belong to a class of higher-plant-specific transcription factors. LBD proteins usually include the highly conserved lateral organ boundaries (LOB) domains that play essential roles in plant growth and response to biotic stresses. However, few studies have addressed the biological functions of LBD genes associated with maize response to drought. Here we cloned the *ZmLBD2* gene from maize and described its role in combating drought. Investigating ZmLBD2 subcellular localization, we show that it localizes to the cell nucleus and can specifically bind with inverted repeats of “GCGGCG”. Under drought stress, *Arabidopsis thaliana* overexpressing ZmLBD2 performed better than the wild-type plants in terms of seed germination rates, root length, relative water content, fresh weight, chlorophyll content, proline content, and antioxidant enzyme content. Arabidopsis overexpressing ZmLBD2 contained less MDA, H_2_O_2_, and ${\text{O}}_{2}^{-}$ than the wild-type plants. Our protein-protein interaction results indicate an interaction between the *ZmLBD2* and *ZmIAA5* genes. In conclusion, the *ZmLBD2* gene positively regulates H_2_O_2_ homeostasis in plants, strengthening drought resistance.

## Introduction

Maize (*Zea mays* L.) is the world’s number one food crop, with strong adaptability and wide cultivation areas across the globe. Drought stress is one of the common factors reducing crop yield. Agricultural economic losses increase year on year as drought rages worldwide. In recent years, a series of genes crucial to combat drought stress has been described in plants (Sahebi et al., 2018; Liu et al., 2020; Wei et al., 2021; Chen et al., 2021; Wang B. et al., 2022). Reactive oxygen species (ROS), mainly including superoxide anion radicals ( ${\text{O}}_{2}^{-}$ ), hydroxyl ions (OH^-^), hydroxyl radicals (·OH), and hydrogen peroxide (H_2_O_2_), accumulate under drought stress and work in various ways to influence the growth of plants and their responses to environmental stresses (Wang et al., 2018; Xiong et al., 2022). Yuan et al. (2019) found that the overexpression of the *NAC066* gene could significantly enhance the tolerance of rice (*Oryza sativa* L.) against drought and oxidative stress by reducing the accumulation of ROS. Hu et al. (2020) reported that TaCDPK13 could be considered the upstream regulatory factor of TaNOX7 to regulate the generation of ROS in wheat (*Triticum aestivum* L.). Xiong et al. (2018) reported that the natural mutation of the *OsLG3* gene strengthened the resistance of rice against drought by inducing the removal of ROS.

The lateral organ boundaries domain (LBD) proteins belong to a class of plant-specific transcription factors, including the conserved lateral organ boundaries (LOB) domain. This domain consists of the highly conserved C motif (C-block), Gly-Ala-Ser motif (GAS-block), and leucine zipper-like motif (leucine zipper-like block). Most LBD family members attribute to the class I, which is mainly responsible for several biological reactions in higher plants, such as growth and responses to adversities (drought, salt, cold, etc.).There are still many unanswered questions about the *LBD* gene. For example, does the *LBD* gene function in other species of plants, and what function(s) does it play? Xiong et al. (2022) found that the ZmLBD5 of maize could increase the tolerance of *Arabidopsis thaliana* against drought by inhibiting the accumulation of ROS. Liu et al. (2020) discovered that the knockout of the *SlLBD40* gene could promote the drought tolerance of tomatoes (*Solanum lycopersicum* L.). Liu et al. (2019) illustrated that the overexpression of *StLBD2-6* and *StLBD3-5* genes might help maintain a normal metabolism for potato (*Solanum tuberosum* L.) to improve its ability to resist drought. Research published by Guo et al. (2020) stated that drought stress induced the ABA(abscisic acid) signal to promote the expression of the *LBD15* gene in *A. thaliana*, which enhanced its tolerance against water stress. Ariel et al. (2010) found that overexpression of *Mt LBD1* could control the structure of *Medicago truncatula* roots under salt stress. Wang Z. et al. (2021) found that the lateral organ boundaries domain (LBD) genes, a plant-specific transcription factor family, play crucial roles in controlling plant architecture and stress tolerance. Wang J. et al. (2021) found that *RrLBD12c*, *RrLBD25*, *RrLBD39*, and *RrLBD40* of Rosa may be potential regulators of salt stress signaling. Ba et al. (2016) reported that *MaLBD5* and *MaJAZ1* might act antagonistically concerning MeJA-induced cold tolerance of banana fruit.

In previous studies, 44 LBD transcription factors have been identified in the maize genome (Zhang et al., 2014). However, only the *ZmLBD5* gene (Zm00001d032286) has been functionally verified regarding the response to drought. The functions of other *ZmLBD* genes remain unclear. In this study, we analyzed how the *ZmLBD2* gene responded to drought. The overexpression of ZmLBD2 in the transgenic *A. thaliana* could improve its drought tolerance by reducing the accumulation of ROS. Our results show that ZmLBD2 can mediate the response of maize seedlings to drought by regulating the homeostasis of hydrogen peroxide, which may positively affect plants’ drought tolerance.

## Materials and methods

### Plant materials and growth conditions

The wild-type (ecotype: Col-0) and transgenic *A. thaliana* (OE2 and OE3) seeds were disinfected with 75% alcohol for 1 min. After surface sterilization with 1% NaClO_3_ for 10 min, they were washed four times with sterile water. Following the disinfection treatment, all seeds were cultivated in 1/2 Murashige and Skoog (MS) media at 4°C in the dark for 3 days. Next, the culture media were moved to a plant incubator (at 22°C with a 16 h light/8 h dark cycle) for the seeds to grow. Two weeks later, the seedlings were transplanted into the soil and grown in a phytotron (at 22°C with a 16 h light/8 h dark cycle). They were treated with 8% PEG6000 (polyethylene glycol 6000) at the three-leaf stage, and the physiological data were measured. We collected their leaves at 0, 6, 12, 24, and 48 h and froze them in liquid nitrogen immediately after the collection to extract their RNA.

### Gene isolation and sequence analysis

The maize *LBD2* gene was obtained from MaizeGDB (https://www.maizegdb.org/) (accessed on 2 June 2022). Homologous sequences of ZmLBD2 were retrieved from the Phytozome database (https://phytozome-next.jgi.doe.gov/) (accessed on 2 June 2022). Amino and multiple sequence alignments were constructed with ClustalX. Our laboratory completed the transcriptome data of maize under different drought treatment times, and the transcriptome data has been uploaded to the NCBI (National Center for Biotechnology Information) database (PRJNA793522).

### The subcellular localization of ZmLBD2

To explore the possible subcellular location of ZmLBD2, the full-length gene coding region of ZmLBD2 was obtained from the TA cloning vector containing the *ZmLBD2* gene fragment. The amplified product was then fused to the CaMV35S promoter and inserted upstream of the coding region of the pCAMBIA1302-eGFP subcellular localization vector. The constructed vector pCAMBIA1302-ZmLBD2-eGFP was introduced into tobacco leaves using agrobacterium-mediated methods. The leaves were then examined at 45 h after infiltration using an Olympus confocal laser scanning microscope (DAPY Olympus, Tokyo, Japan). The primers used here are listed in **Supplementary Table S1**.

### Induced expression of ZmLBD2 protein and detection by Western blotting

Full-length cDNA of ZmLBD2 was amplified and inserted into a pET28a vector driven by a Cauliflower Mosaic Virus 35S (CaMV35S) promoter. The constructed vector pET28a-ZmLBD2-His was transformed into BL21 *E. coli* competent cells. Then, the protein expression of the positive recombinant plasmid was performed under isopropyl β-D-thiogalactoside (IPTG) induction conditions, and finally, the protein content was detected by SDS-PAGE (polyacrylamide gel electrophoresis) electrophoresis and Western blotting.

### Analysis of DNA binding characteristics of ZmLBD2

First, the cDNA of the target gene (*ZmLBD2*) was constructed on the pGADT7 empty vector to form the recombinant vector pGAD-ZmLBD2. Three tandem repeats of GCGGCG were synthesized by artificial synthesis to construct the yeast monohybrid reporter vector pHIS2-TCS. To verify the specificity of ZmLBD2 for this target recognition, we synthesized three tandem repeated mutant targets, GAGGAG. *EcoR* I and *Sac* I restriction sites was added at both ends to construct the yeast monohybrid reporter vector pHIS2-mTCS. Finally, the binding site of the *ZmLBD2* gene was verified by yeast one-hybrid (the yeast one-hybrid strain used in this study was Y187).

### Analysis of the drought resistance of the *ZmLBD2* gene in yeast

We designed the yeast plasmid vectors pYES2-ZmLBD2 and transformed them into competent INVSc1 yeast strains by the yeast transformation method. We cultivated the transformed strain in YPD (Yeast Extract Peptone Dextrose) liquid media. When the OD_600_ (optical density 600) concentration reached 0.8 after shake culture, we collected the strain bodies by centrifugation and dissolved them with physiological saline. After diluting the solution into six gradients, we added 5uL of each gradient into YPD culture media with and without mannitol.

### RNA extraction and quantitative RT-qPCR analysis

TRIzol reagent (TAKARA BIO INC. Dalian, China) was used to extract total RNA from the maize (maize inbred lines H8186) treated under different stresses and from the *A. thaliana* seedlings. After being identified, the RNA was used to generate cDNA with TaKaRa reverse transcription kit. And SYBR GreenFastqPCRMix kit (ABclonal Biotechnology Co., Ltd, RM21203) was used for qPCR amplification. The reaction procedure was as follows: denaturation at 95°C for 10 min, followed by 40 cycles of denaturation at 95°C for 10s, at 55°C for the 40s, at 72°C for 20s, and 72°C for 4 min. Zm18S and AtACTIN8 were selected as the internal reference genes for maize and *A. thaliana*, respectively. The 2^−ΔΔCT^ method was used to analyze the relative expression levels of relevant genes (Jiao et al., 2022; Wang C. et al., 2022). All primers used in this study are listed in **Supplementary Table S1**.

### The genetic transformation of *A. thaliana* and phenotypic analysis

In this study, we constructed the plant overexpression vectors pCAMBIA3301-ZmLBD2-bar and transformed the recombinant plasmids into *Agrobacterium tumefaciens* strain GV3101; and then we infected *A. thaliana* with the strain by floral dip method (Noman et al., 2019). We cultivated the *A. thaliana* in 1/2 Murashige and Skoog (MS) media, screened out the transgenic plants of T0 generation and collected their leaves for PCR and test strip assay. Our research used the seeds of representative homozygous *A. thaliana* OE2 and OE3 of T3 generation (Transgenic generation 3).

To examine the responses of the transgenic and wild *A. thaliana* to drought, we disinfected both the seeds of the wild type and the transgenic one with the *ZmLBD2* gene (OE2 and OE3) with 75% alcohol for 1 min. After conducting surface sterilization with 1% NaClO_3_ for 10 min, we washed the seeds four times with sterile water. We cultivated the seeds in 1/2MS media and performed drought experiments by adding 200 and 300 mM mannitol. After cultivation at 4°C for 3 days, they were moved to another environment and grew at 24°C and 80% relative humidity with a 16 h light/8 h dark cycle for 5 days. Then, we determined their germination rates and root length. The main root length was measured using the DJ-GXG02 root image analysis system (DianJiang Technology; http://www.dianjiangtech.com/). Each sample contains three biological replicates. One-way ANOVA method was used for statistical analysis. To identify the survival rates, we transplanted the *A. thaliana* seedlings, which sprouted in the 1/2MS media into the soil, and cultivated them at 22°C and 50% relative humidity with a 16 h light/8 h dark cycle for 3 weeks. After that, we stopped watering them for 14 days and took some pictures. Fourteen days later, we re-watered them for 3 days and calculated their survival rates (Ju et al., 2020).

### Tetranitroblue tetrazolium chloride (NBT) staining, diaminobenzidine (DAB) staining, and oxidative stress analyses

Tetranitroblue tetrazolium chloride (NBT) and diaminobenzidine (DAB) were used to stain the seedling leaves of the wild-type and transgenic *A. thaliana* (OE2 and OE3) after drought treatment to examine the accumulation of superoxide anion ( ${\text{O}}_{2}^{-}$ ) and Hydrogen peroxide (H_2_O_2_) (Zhao J. Y. et al., 2020). The spectrophotography was used to test the activity of catalase (CAT), superoxide dismutase (SOD), malondialdehyde (MDA), and peroxidase (POD) (Zhao Q. et al., 2020; Ju et al., 2020; Liu et al., 2021; Li et al., 2021). The weighing method was used to determine relative water content (Sapes and Sala, 2021). The methods mentioned above were used to evaluate the concentration of hydrogen peroxide and the amount of ${\text{O}}_{2}^{-}$ .

### Yeast two-hybrid system

The CDS of the *ZmLBD2* gene was cloned into the c-terminus of the GAL4 DNA-binding domain of the pGBKT7 vector. We used the recombinant plasmid ZmLBD2-BD as a decoy to search for every potential interaction between the encoded proteins on the STRING database (https://string-db.org/), and we cloned the CDS of interactive candidate gene *ZmIAA5* into the pGADT7 vector. The recombinant plasmids ZmLBD2-BD and ZmIAA5-AD were then transformed into yeast cells AH109 using the lithium acetate method (Xu et al., 2007). The transformation efficiency was examined on DDO culture medium SD (synthetic-defined)/-Trp/-Leu, and the protein-protein interaction was verified on QDO medium SD/-Leu/-Trp/-His/-Ade. pGADT7-T and pGBKT7-53 were used as positive controls, and pGADT7-T and pGBKT7-lam were used as negative controls. The yeast transformation procedure was completed concerning the yeast transformation system produced by Beijing Kulaibo Technology Co., Ltd.

### Bimolecular fluorescence complementation

The ORFs (Open Reading Frames) of ZmLBD2 and ZmIAA5 were cloned to the n-terminus and c-terminus of the coding regions of yellow fluorescent proteins (YFP) to construct plasmids nYFP-ZmLBD2 and cYFP-ZmIAA5. Five microliters of each of the two recombinant plasmids were co-transformed into tobacco mesophyll cells by agroinfiltration (Yu et al., 2021). The leaves were then examined at 45 h after infiltration using an Olympus confocal laser scanning microscope (DAPY Olympus, Tokyo, Japan).

### Pull-down assay

We constructed Flag-ZmIAA5 and Myc-ZmLBD2 vectors and purified the fusion proteins by immunoprecipitation using Anti-Flag M1 Affinity Gel. And we purified the Myc-ZmLBD2 recombinant proteins using amylose resin (Park et al., 1998). Flag or Flag-ZmIAA5 was added with equal amounts of Myc-ZmLBD2 protein beads into the buffer (50 mmol/L Tris-HCl, pH 8.0, 100 mmol/L sodium chloride, 0.2% glycerol and 0.5% TritonX-100) and incubated at 4°C for 6 h. The protein complexes were eluted by boiling the beads with electrophoresis loading buffer. Ultimately, the crude extracts and eluted products were determined on 10% SDS-PAGE, and the chemiluminescence signal was detected by Western Blot using Flag-tagged antibodies.

### Statistical analysis

Data were tested by analysis of variance using SPSS software (SPSS USA). The data are the mean ± standard deviation (SD) of three biological replicates. The significance was analyzed using Student’s t-tests. The * and ** represents p < 0.05 and p < 0.01, respectively. The figures were prepared with GraphPad Prism 7.

## Results

### Analysis of ZmLBD2 sequence and expression pattern

**Figure 1:**
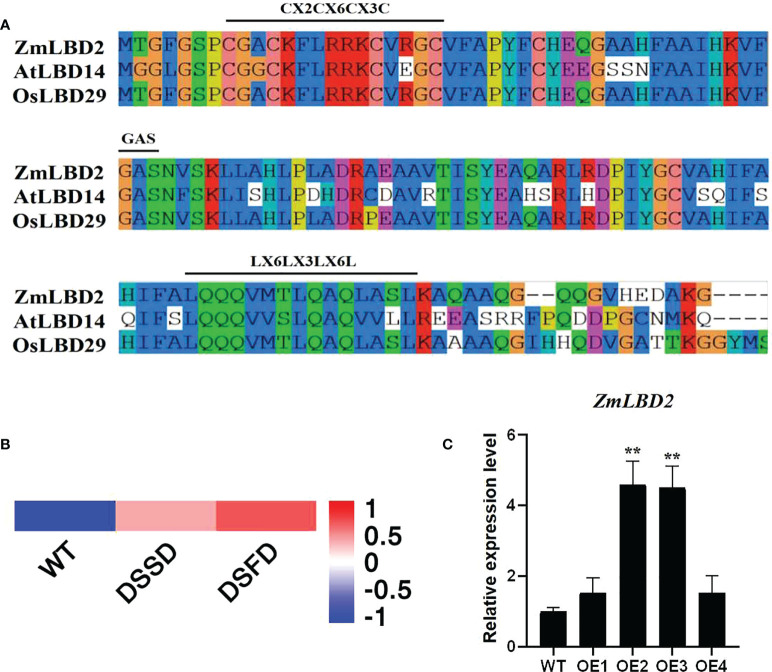
Sequence and expression pattern analysis of ZmLBD2. **(A)** LOB domain sequence alignment of ZmLBD2 and LBD members from Arabidopsis and *Oryza sativa*. The class-I LBD members had typical CX2CX6CX3C, GAS, and LX6LX3LX6L domains. The alignment was performed using DNAMAN software. **(B, C)** The expression of ZmLBD2 upon drought treatment in maize. Total RNA was isolated from 3-leaf seedlings grown without (0 h) or with 8% PEG6000 treatment. Transcript levels of ZmLBD2 were determined by qPCR, using Zm18S as reference genes. The ** represents p < 0.01.

In this study, we cloned the *ZmLBD2* gene from maize from the H8186 selfing line. ZmLBD2 has a full-length CDS of 831bp, which encodes a polypeptide consisting of 244 amino acid residues with a predicted molecular weight of 25.47kD and a pI value of 5.66 (**Table 1**). The results of sequence alignment show that the ZmLBD2 contains CX2CX6CX3C, a typical conserved DNA-binding domain. GAS and leucine zipper-like motifs (LX6LX3LX6L) are primarily used to distinguish the members of the maize LBD family into class I and class II (**Figure 1A**).

**Table 1:** The physiochemical characteristics of maize ZmLBD2 proteins.

Gene name	Gene ID	CDS length (bp)	Protein length (aa)	MW (KDa)	pI
*ZmLBD2*	Zm00001d027679	831	244	25.47	5.66

We drew a heatmap (**Figure 1B**) based on the transcriptome data of maize roots (PRJNA793522) with different drought treatment processing times. The response of ZmLBD2 to drought stress was studied by RT-qPCR using maize plants under 8% PEG6000 drought treatment. The expression of the *ZmLBD2* gene was highly induced as the time of suffering drought stress increased (**Figure 1C**), indicating that the *ZmLBD2* gene plays a crucial role in responding to drought.

### Subcellular localization of ZmLBD2 protein in tobacco

**Figure 2:**
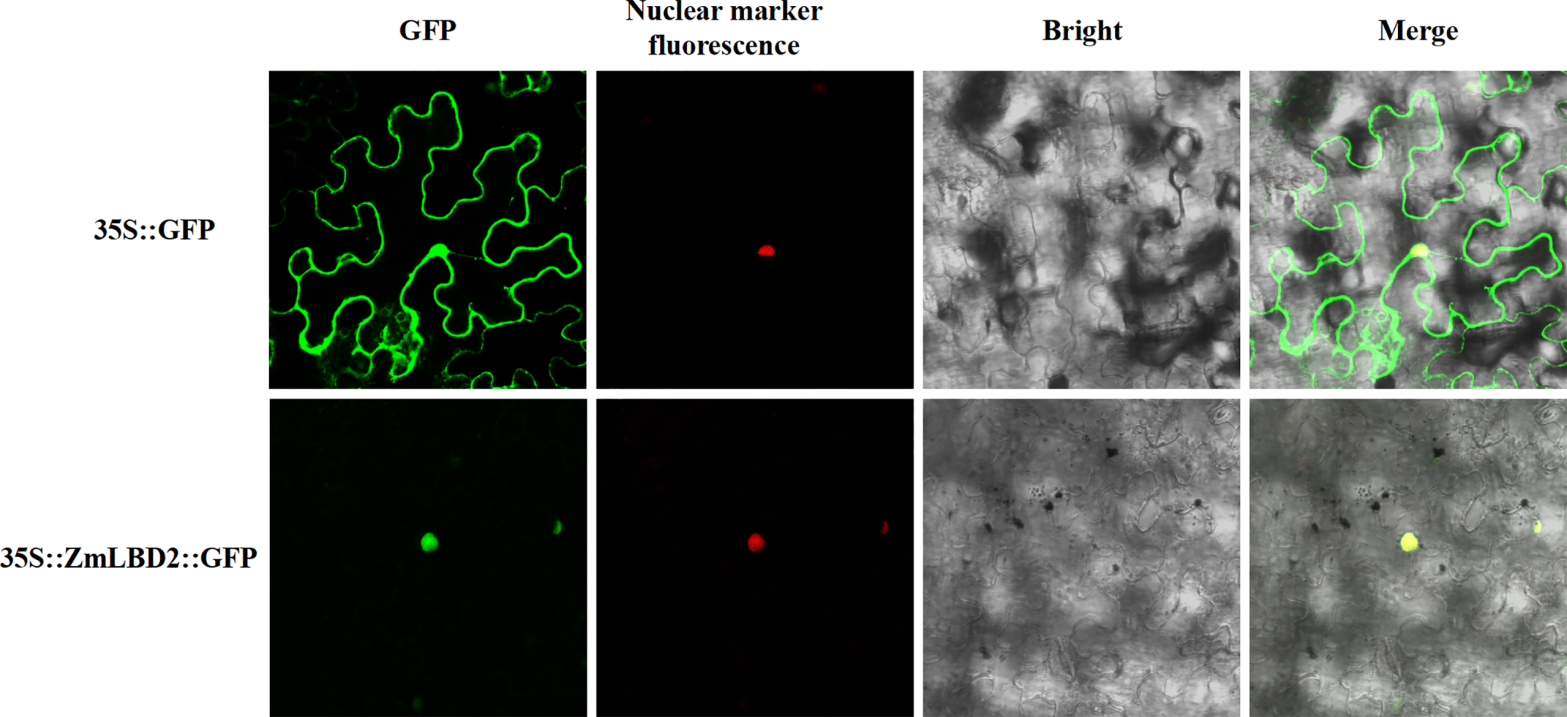
Subcellular localization analysis of ZmLBD2 proteins in tobacco cells. The scale bar represents 50 μm.

We designed subcellular localization vectors for the *ZmLBD2* gene and transformed them into tobacco mesophyll cells *via Agrobacterium tumefaciens*. By confocal microscope (Olympus, Japan), we observed that the proteins transcribed from the *ZmLBD2* gene localized to the cell nucleus (**Figure 2**).

### Efficient expression of ZmLBD2 in a prokaryotic system

**Figure 3:**
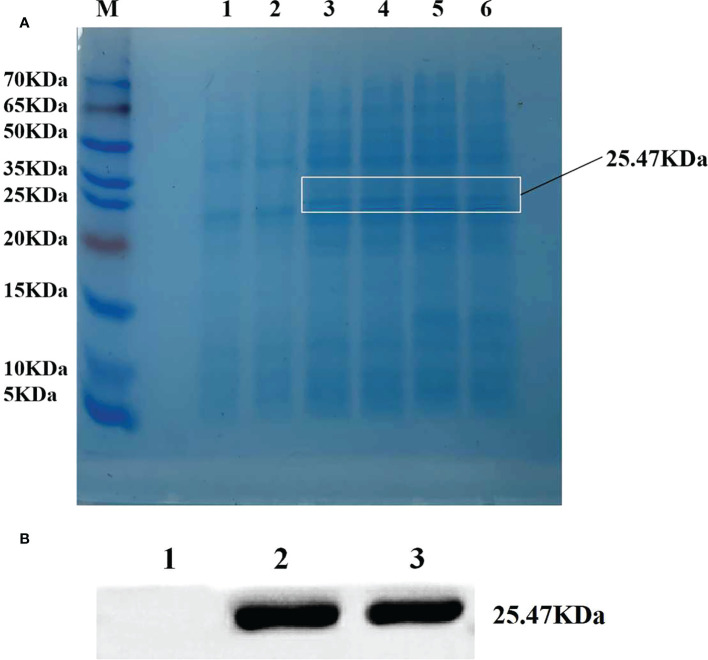
Efficient expression of ZmLBD2 in a prokaryotic system. **(A)** SDS-PAGE electrophoretic analysis of ZmLBD2 recombinant protein. M: Protein marker; 1: pET22b empty vector was not induced by IPTG; 2: pET22b-ZmLBD2 non-induced bacteria; 3-6: pET22b-ZmLBD2 was produced by IPTG for 4 h. **(B)** Western blotting detection of ZmLBD2 protein; 1: pET22b empty vector; 2-3: pET22b-ZmLBD2 after inducing.

BL21 strain with pET28a-ZmLBD2 recombinant plasmids was selected for single-colony shake cultivation. When the OD600 concentration of the cultures was between 0.6 and 1.0, IPTG with a final concentration of 0.1 mM was added to induce protein expression. After that, BL21 strain with transformed pET28a-ZmLBD2 recombinant plasmids was obtained. We induced the expression at 26°C for 4 h and observed the changes in protein expression. The SDS-PAGE results showed that a clear band around 25.47 kDa was spotted for the recombinant protein of the ZmLBD2, which indicated that the ZmLBD2 recombinant protein could be expressed stably and effectively in the BL21 strain with the induction of IPTG (**Figure 3A**). The Western blot results showed that a specific band could be seen clear between 25 kDa and 35 kDa (**Figure 3B**), indicating that the ZmLBD2 protein of maize was successfully induced and expressed *in vitro*.

### The DNA binding characteristics of ZmLBD2

**Figure 4:**
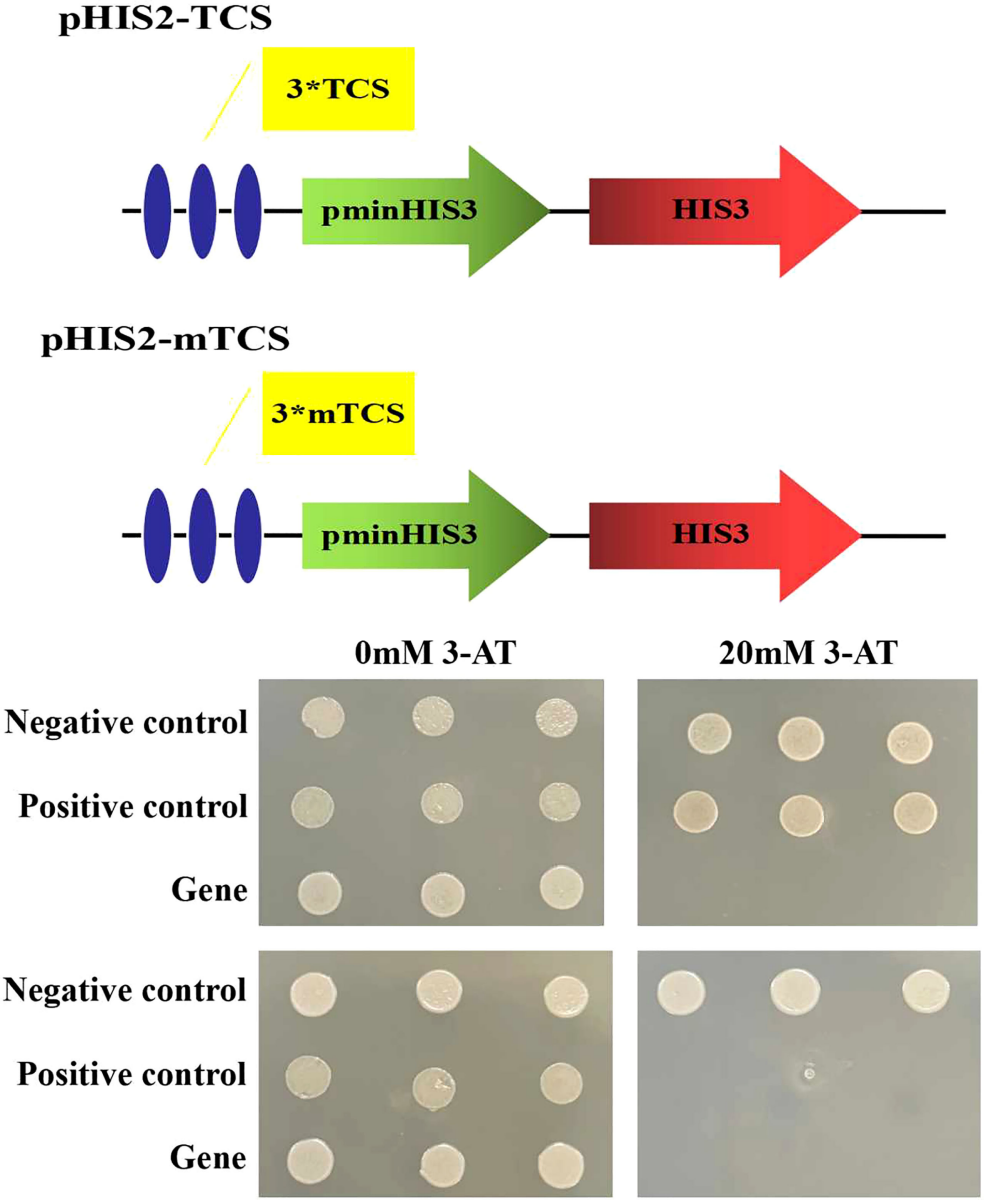
DNA-binding assays of ZmLBD2 in Y1HGold yeast cells. Yeast cells were assayed on SD/Trp-/Leu-/His- medium; Negative control: p53HIS2+p GAD-ZmLBD2; Positive control: p53HIS2+pGAD-Rec2-53p53; Gene: different positive colonies; TCS: GCGGCG; mTCS: GAGGAG; 3*: The sequence was repeated three times; 3-AT: 3-amino-1,2,4-triazole.

On the 20 mM 3-AT SD/Trp-/Leu-/His triple deficiency culture plate, only the yeast strains co-transformed with pGADT7-ZmLBD2 and pHIS2-TCS and the positive control p53HIS2+pGAD-Rec2-p53 can grow normally (**Figure 4**), indicating that ZmLBD2 can activate the expression of the reporter gene HIS by binding to the “GCGGCG” inverted repeat sequence. To verify the specificity of the DNA binding method, the pGADT7-ZmLBD2 and pHIS2-mTCS vectors were co-transformed into yeast cells on the SD/Trp-/Leu-/His- triple lack culture plate containing 20 mM 3-AT. Only the positive control could grow normally. The binding of the *ZmLBD2* gene to the “GCGGCG” inverted repeat is specific.

### Dimer-forming ability of ZmLBD2

**Figure 5:**
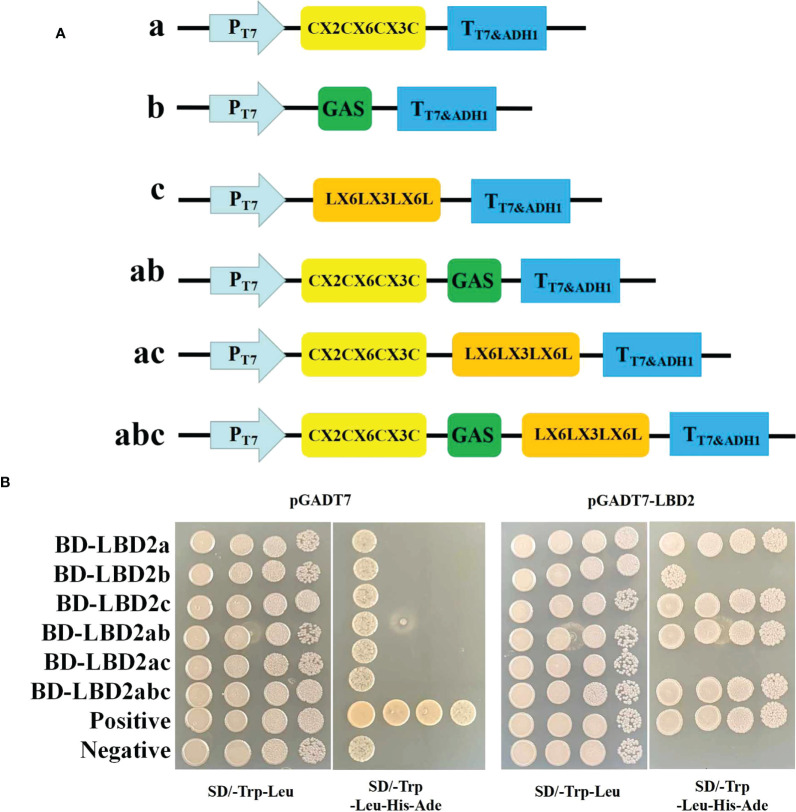
Analysis of the dimer-forming ability of ZmLBD2. **(A)** Strategy for yeast two-hybrid vector construction of the maize ZmLBD2 sequence. **(B)** The ability of ZmLBD2 to form dimers in the yeast strain Y2H Gold. The Y2H Gold strains containing target plasmids were diluted and cultured on SD/-Trp-Leu media or SD/-Trp-Leu-His-Ade media. Fragments a, b, and c represent CX2CX6CX3C, GAS, and LX6LX3LX6L.

To test the dimerization ability of ZmLBD2, six truncated peptide fragments (a, b, c, ab, bc, and abc) of ZmLBD2 were tested for interaction with ZmLBD2. Fragments A, B, and C represent the N-terminal CX2CX6CX3C, the GAS and LX6LX3LX6L coiled-coil motifs, and the C-terminal domain, respectively (**Figure 5A**). To further define the necessary regions for ZmLBD2 interaction, we screened for homo- and heterodimerization by yeast two-hybridization (**Figure 5B**).

### Identification of the drought tolerance of transgenic yeast with ZmLBD2

**Figure 6:**
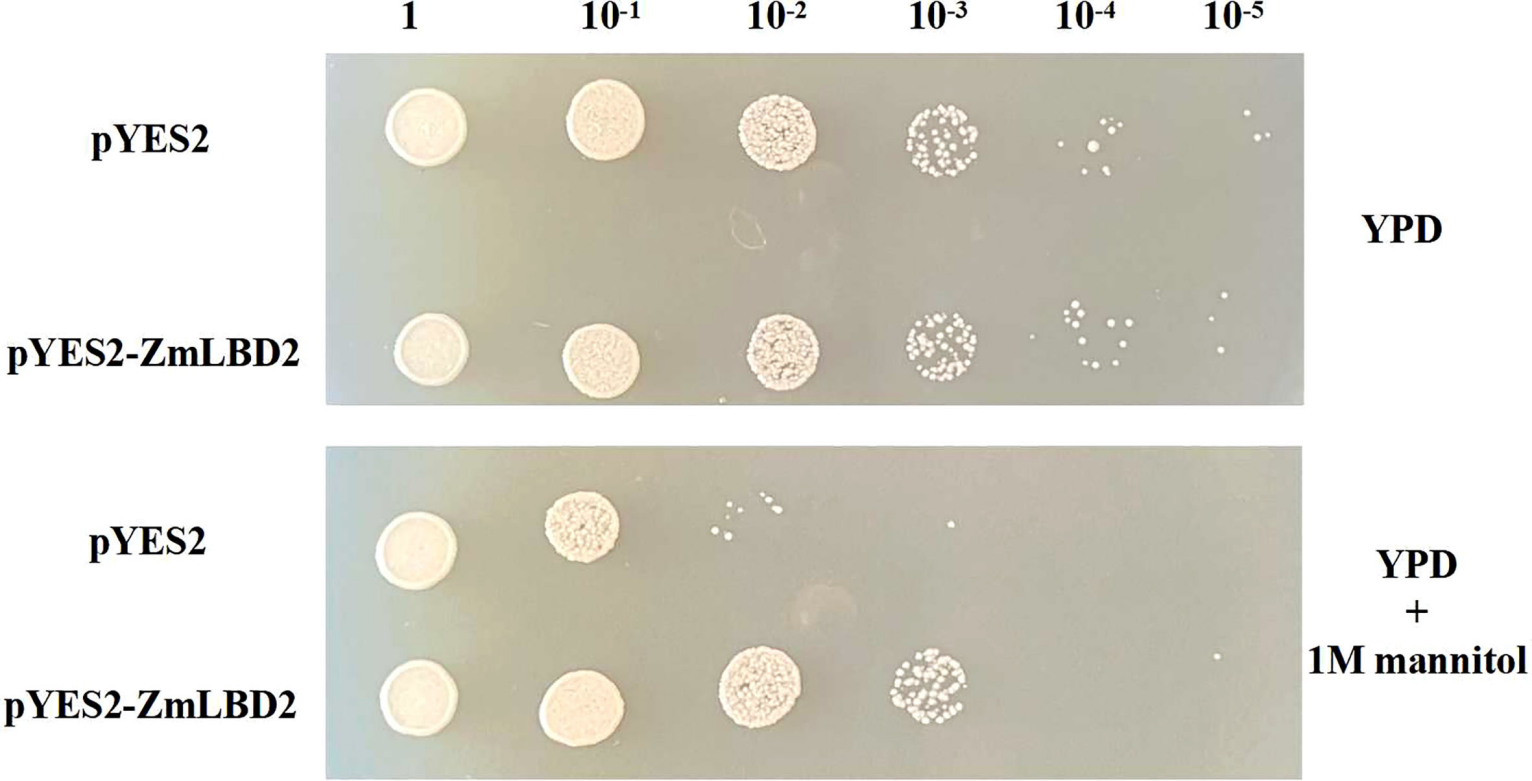
Phenotypic analysis of ZmLBD2 transgenic yeast strains under mannitol stress.

We detected no differences between transgenic yeast with pYES2 empty vector and that with *ZmLBD2* gene in regular YPD culture media (**Figure 6**). But, in the YPD culture media containing 1M of mannitol, the transgenic yeast with ZmLBD2 grew better than that the pYES2 empty vector. This tentatively indicates that the *ZmLBD2* gene has the power to resist drought.

### Genetic transformation and molecular identification of the *ZmLBD2* gene in *A. thaliana*

**Figure 7:**
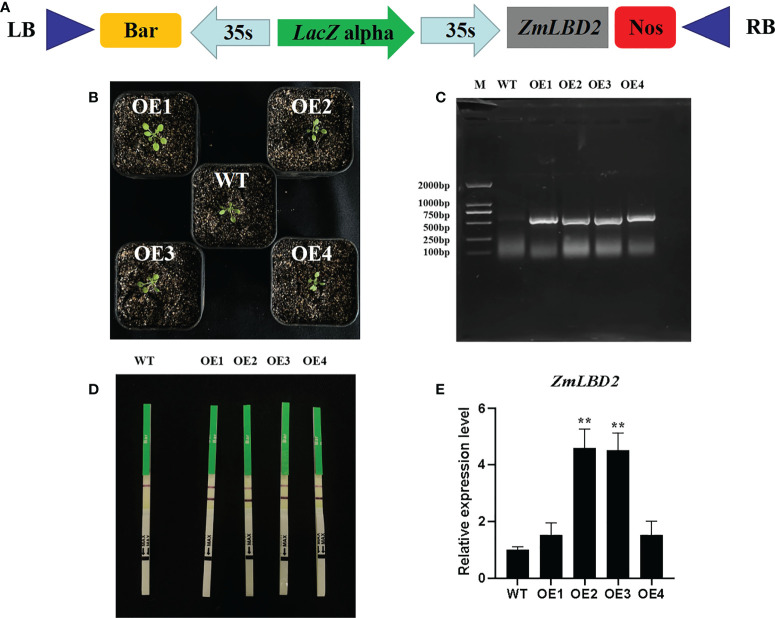
Identification of Arabidopsis overexpressing the *ZmLBD2* gene. **(A)** Schematic of expression vectors pCAMBIA3301-ZmLBD2-Bar. **(B)** Transgenic Arabidopsis lines. **(C)** The PCR detection of the *Bar* gene in leaves of wild type (WT) and transgenic lines. **(D)** Rapid test strip analysis of the *Bar* gene in wild-type (WT) leaves and transgenic lines. **(E)** qRT-PCR analysis of the *ZmLBD2* gene in leaves of wild type (WT) and transgenic lines. The expression level was normalized to that of Arabidopsis AtACTIN8. Data were expressed as the mean of triplicate values, and error represented the SD. The * and ** represents p < 0.05 and p < 0.01, respectively.

In this study, we transformed the plasmid DNA of constructed plant overexpression vector pCAMBIA3301-ZmLBD2-bar (**Figure 7A**) into the EHA105 chemically competent cells and finally, obtained four transgenic positive *A. thaliana* plants of T3 generation by using floral dip method (**Figures 7B-D**). By qRT-PCR, we found that the *ZmLBD2* gene was expressed in all transgenic plants, but a higher expression was observed in OE2 and OE3 (**Figure 7E**). In conclusion, the transgenic plants OE2 and OE3 could be used to further identify the ability of ZmLBD2 to combat drought.

### Overexpression of ZmLBD2 enhanced drought tolerance in transgenic *A. thaliana*

**Figure 8:**
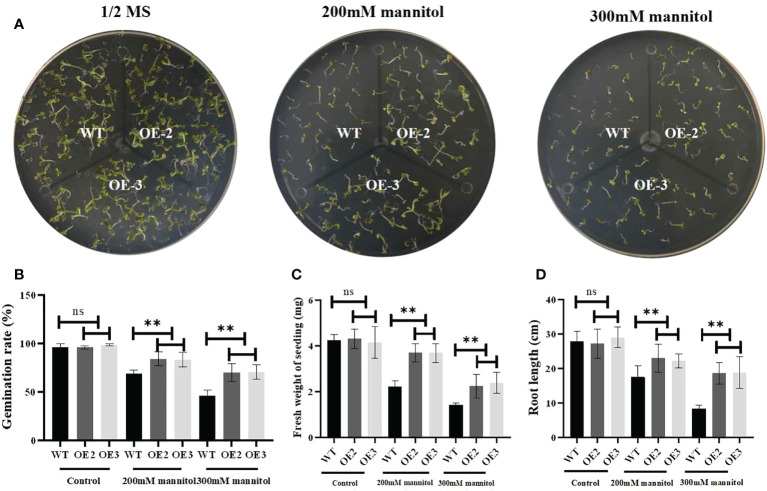
Drought stress responses of ZmLBD2-overexpressing transgenic Arabidopsis and wild-type plants. **(A)** Seedling growth by wild-type and ZmLBD2-overexpressing transgenic Arabidopsis plants under treatment with 0, 200, and 300 mM of mannitol for 5 days. **(B)** Statistical analysis of ZmLBD2-overexpressing transgenic lines and wild-type seed germination rates under 0, 200, and 300 mM mannitol, respectively. **(C)** Statistical analysis of ZmLBD2-overexpressing transgenic lines’ and wild-type’s fresh weight under 0, 200, and 300 mM mannitol, respectively. **(D)** Statistical analysis of ZmLBD2-overexpressing transgenic lines’ and wild-type’s root length under 0, 200, and 300 mM mannitol, respectively. Data were expressed as the mean of triplicate values, and error represented the SD. The ** represents p < 0.01, respectively. Non-significant (ns).

To investigate its biological function, we generated ZmLBD2 overexpressing *A. thaliana*. The seeds of the transgenic plants OE2 and OE3 of T3 generation were cultivated in 1/2 MS media with 0, 200, or 300 mM of mannitol. After cultivation for 5 days, the results of comparing their germination status showed no differences between the wild type and the transgenic *A. thaliana* in the 1/2 MS media with 0 mannitol in terms of germination. As the concentration of mannitol increased, however, the OE2 and OE3 grew better than the wild type did (**Figure 8A**). Moreover, the germination rates (**Figure 8B**) and fresh weight (**Figure 8C**) and root length (**Figure 8D**) of the OE2 and OE3 were much higher than that of the wild type as the concentration of mannitol increased.

**Figure 9:**
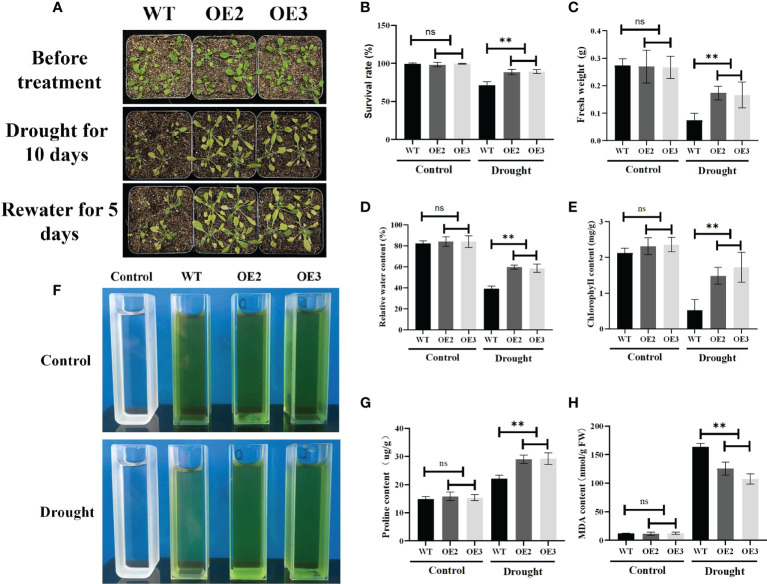
Phenotypes of the ZmLBD2-overexpressing transgenic plants and wild-type controls in *A. thaliana* under drought conditions. **(A)** Drought treatment using 30 days plants. The wild-type controls and transgenic plants were grown in pots for 30 days and then subjected to drought treatment for 10 days. The plants were watered again 5 days after the treatment. Survival rate **(B)**, fresh weight **(C)**, relative water content **(D)**, chlorophyll content **(E, F)**, proline content **(G)**, and MDA content **(H)** in transgenic Arabidopsis plants and wild-type controls under normal conditions and drought stress. Data were expressed as the mean of triplicate values, and error represented the SD. The ** represents p < 0.01, respectively. Non-significant (ns).

To further study the functions of ZmLBD2 in response to drought stress, we transplanted the 5-day-old cultivated plants into the soil. Then, we conducted drought treatment on 30-day-old *A. thaliana* for 10 days. After re-watering for 5 days, we found that the OE2 and OE3 performed much better than the wild type in terms of survival rates (**Figures 9A, B**), fresh weight (**Figure 9C**), relative water content (**Figure 9D**), chlorophyll content (**Figures 9E, F**), and proline content (**Figure 9G**). OE2 and OE3 also had less MDA than the wild-type plants (**Figure 9H**). These results indicate that the overexpression of ZmLBD2 can significantly boost the drought resistance of *A. thaliana*.

### Reducing the accumulation of reactive oxygen species by the overexpression of the *ZmLBD2* gene under drought stress

**Figure 10:**
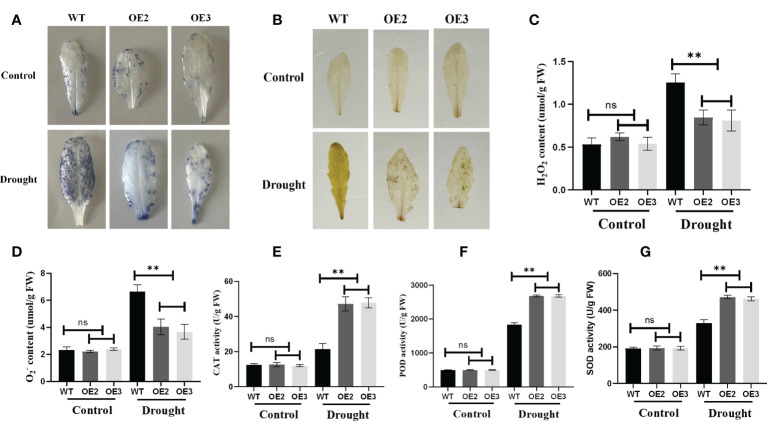
Reactive oxygen species staining and physiological indices in ZmLBD2 transgenic Arabidopsis. **(A)** NBT staining of leaves for H_2_O_2_ from ZmLBD2 transgenic seedlings and wild-type plants under normal conditions and drought stress (30 days seedlings were subjected to drought for 10 days). **(B)** DAB staining of leaves for H_2_O_2_ from ZmLBD2 transgenic seedlings and wild-type plants under normal conditions and drought stress (30 days seedlings were subjected to drought for 10 days). **(C, D)** H_2_O_2_ and ${\text{O}}_{2}^{-}$ content in leaves from ZmLBD2 transgenic seedlings and wild-type plants under normal conditions and drought stress. CAT activity **(E)**, POD activity **(F)**, and SOD activity **(G)** in leaves from ZmLBD2 transgenic seedlings and wild-type plants under normal and drought conditions. Data were expressed as the mean of triplicate values, and error represented the SD. The ** represents p < 0.01, respectively. Non-significant (ns).

Reactive oxygen species (ROS) are essential signaling molecules for plant growth, which play a crucial role in adapting to environments. Therefore, we analyzed the ROS levels of wild and transgenic plants by evaluating the accumulated amount of hydrogen peroxide and superoxide under drought stress. After staining with Nitrotetrazolium Blue chloride (NBT) and Diaminobenzidine (DAB), we found that the accumulation of hydrogen peroxide and superoxide of the wild type was more than that of the OE2 and OE3 (**Figures 10A, B**). Meanwhile, we compared the accumulation and production of hydrogen peroxide and O^2−^ in the leaves of the wide type OE2 and OE3 maize seedlings. Hydrogen peroxide and superoxide anion accumulated less in the leaves of seedlings with the overexpression of ZmLBD2 than in wild-type seedlings (**Figures 10C, D**). SOD, CAT, and POD enzymes directly eliminate ROS to regulate ROS levels in plant cells. They turn the ROS into less active and less toxic hazardous substances. We found that, with the threat of drought, SOD, CAT, and POD enzymes were more active in the *A. thaliana* with the overexpression of ZmLBD2 than those in the wild type (**Figures 10E-G**).

### The effects of ZmLBD2 on the expression of drought-stress-responsive genes

**Figure 11:**
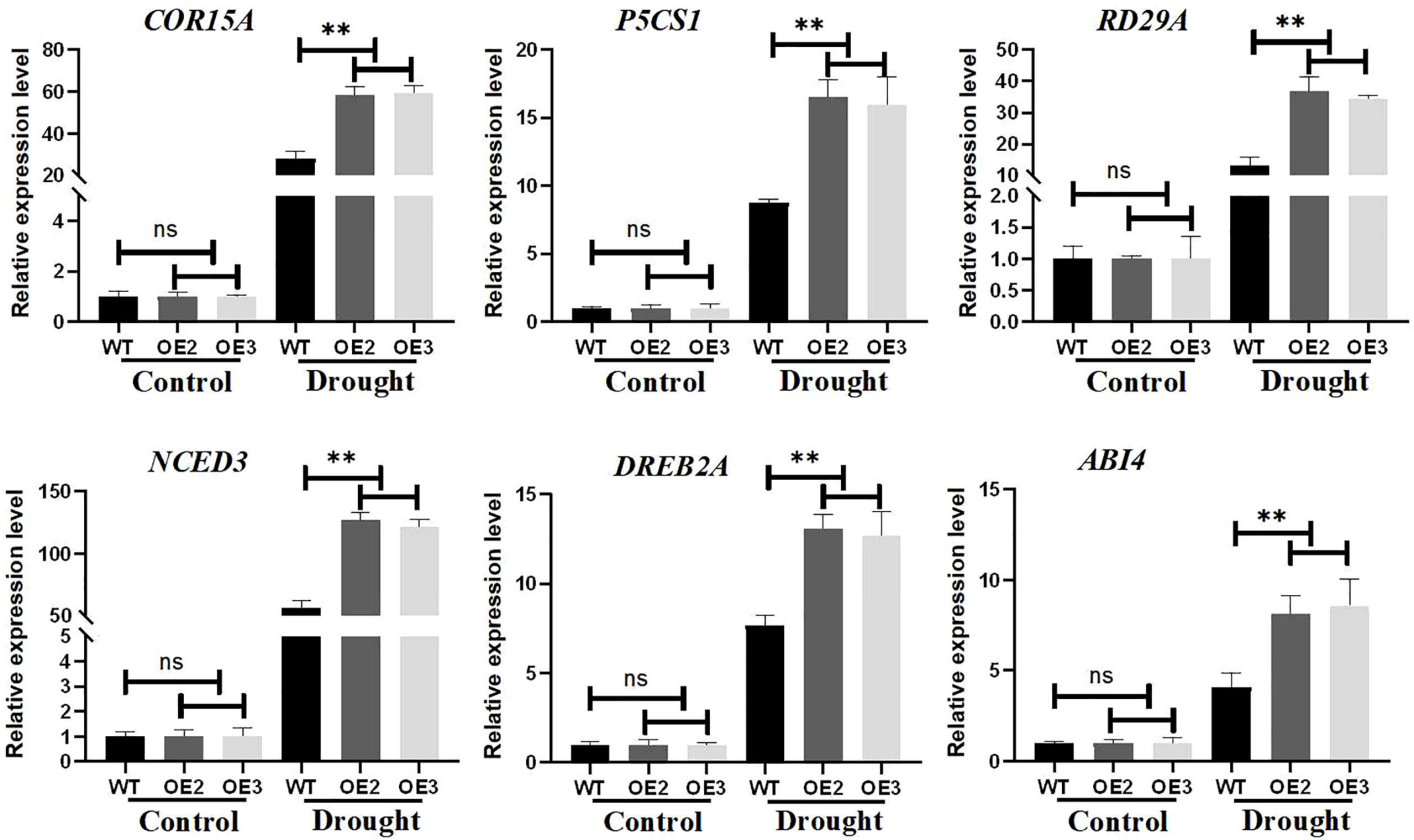
Expression levels of drought-stress-related genes in ZmLBD2 transgenic Arabidopsis. Total RNA was isolated from 15-day-old seedlings grown without (CK) or drought treatment for 10 days. Transcript levels of COR15A, P5CS1, RD29A, NCED3, DREB2A, and ABI4 in the transgenic lines and wild type were determined by qPCR AtACTIN8 as reference genes. Fold change was calculated by 2^−ΔΔCT^. The ** represents p < 0.01, respectively. Non-significant (ns).

To investigate the molecular mechanism of ZmLBD2 regulation under drought conditions, we performed a quantitative expression analysis of six stress-related genes in transgenic Arabidopsis and wild-type plants. Under normal growth conditions, the transcript expression levels of P5CS1, RD29A, COR15A, NCED3, DREB2A, and ABI4 were not significantly different between wild-type control and overexpressing lines. However, under drought conditions, the expression of these genes was rapidly induced and at higher levels, and the expression levels of these genes in ZmLBD2-overexpressing seedlings were lower than in wild-type controls (**Figure 11**). Therefore, ZmLBD2 may positively regulate the expression of drought stress-related genes in plants under drought stress.

### *ZmIAA5*, a candidate *ZmLBD2* interacting gene

**Figure 12:**
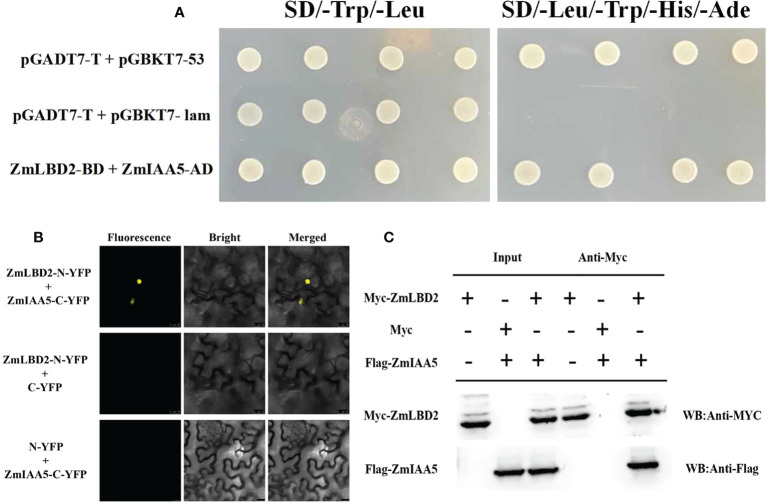
Interaction between ZmLBD2 and ZmIAA5. **(A)** Interaction analysis of ZmLBD2 and ZmIAA5 using a yeast two-hybrid system. **(B)** Interaction of ZmLBD2 and ZmIAA5 as determined with a BiFC assay. **(C)** Pull-down assay ofZmLBD2 and ZmIAA5.

To study how the *ZmLBD2* gene influences the drought resistance of the transgenic *A. thaliana*, we screened a cDNA library for maize in a yeast two-hybrid system (with ZmLBD2 as a decoy) and identified an interactive candidate gene ZmIAA5 (Zm00001d043878). **Figure 12A** shows that all yeast grew normally in DDD culture media (SD/-Trp/-Leu), whereas only that with the coexpression of AD-ZmIAA5 and BD-ZmLBD2 grew well in QDO selective media (SD/-Trp/-Leu/-His/-Ade). We also conducted a BiFC assay to corroborate the interaction between ZmLBD2 and ZmIAA5. When nYFP-ZmLBD2 and cYFP-ZmIAA5 were coexpressed, we detected the fluorescence from yellow fluorescent proteins (YFP) in the nuclei of tobacco mesophyll cells. However, no fluorescence was detected in terms of the coexpression of nYFP-ZmLBD2 and cYFP, or nYFP and cYFP-ZmIAA5 (**Figure 12B**). Pull-down assay was performed to test the purified recombinants Myc-ZmLBD2 and Flag-ZmIAA5 *in vitro*. The Myc-ZmLBD2 protein interacted with Flag-ZmIAA5 protein, but it did not bind to Myc (**Figure 12C**). These results indicate a direct gene interaction between ZmLBD2 and ZmIAA5.

## Discussion

LBD genes are specific plant transcription factors. They are divided into class I and class II following the differences in the LOB domain. LBD transcription factors from the class I contain the complete LOB domain, and they are mainly responsible for plant growth, such as developing lateral organs, extending leaves, and regeneration (Bell et al., 2012; Liu et al., 2018; Zhang et al., 2020; Huang et al., 2020). Class II LBD proteins hold an incomplete leucine zipper-like domain, so they cannot form the coiled-coil structure. They are primarily responsible for secondary metabolite production in plants, such as the synthesis of anthocyanidin and the growth of roots. They also play an active role in responding to pathogenesis, osmotic stress, salt stress, and drought stress (Lim et al., 2015; Liu et al., 2016; Zhang et al., 2017; Fernando Gil et al., 2018; Liu et al., 2019; Liu et al., 2020; Guo et al., 2020). Our study found that the *ZmLBD2* gene was significantly induced to express under drought.

The growth of plants is not only regulated by internal hormones and transcription factors but also influenced by external environments. When the living environment changes or hazardous factors occur, plants need to respond to the environmental signals from the outside to better adapt to the environment. Liu et al. (2020) reported that the *SlLBD40* gene in tomato (*Solanum lycopersicum* L.) was overexpressed under drought stress, which caused the plant to wither. In addition, the expression of this gene was affected by the signal transmission pathway of jasmonic acid. Huang et al. (2020) found that most *Physcomitrium patens* mosses increased the expression of the *PpLBDs* gene after they were processed by mannitol. They extrapolated that the expression of PpLBDs might strengthen the drought resistance of the mosses. The expression of the *VvLBD1* gene in grape (*Vitis vinifera* L.) increased considerably under the threats of mannitol, salt stress, and heat stress, indicating the significance of VvLBD1 in the responses to environmental signals (Cao et al., 2016). Liu et al. (2019) adopted qRT-PCR assay to potato (*Solanum tuberosum* L.) with drought treatment and found that the expression of StLBD1-5 decreased while that of the StLBD2-6 and StLBD3-5 increased. This finding reveals the connection between these three genes and the response of potatoes to drought stress. The research published by Guo et al. (2020) showed that the *A. thaliana AtLBD15* gene could directly bind to the abscisic acid signaling pathway factor ABI14’s promoter to close the stomas and enhance drought resistance. Our study shows that the overexpression of the maize *ZmLBD2* gene can promote *A. thaliana* seeds to burgeon and grow roots under drought, showing that the ZmLBD2 can strengthen the drought tolerance of plants.

Drought stress is to blame for the production of ROS. To protect cells from oxidative damage caused by oxidative stress when accumulating too much ROS, plants have developed various antioxidant mechanisms to remove toxins caused by excess ROS (McGrann et al., 2015). Xiong et al. (2022) found that the ROS content in Arabidopsis overexpressing zmLBD5 was reduced, and the SOD and POD activities were higher than those in wild-type Arabidopsis. Xing et al. (2020) found that *A. thaliana* overexpressing the *CmLOX10* gene under drought conditions had lower MDA and hydrogen peroxide content than the wild type and had strong drought resistance. In our research, the activity of CAT, SOD, and POD increased in the *A. thaliana* overexpressing ZmLBD2, and the levels of H_2_O_2_ and ${\text{O}}_{2}^{-}$ were higher in the wild type. This finding indicates that the ZmLBD2 can enhance drought resistance by reducing ROS accumulation.

Protein interaction has become a necessary link to further explore the role of maize LBD transcription factor genes in plant growth and development and abiotic stress response. Xu et al. (2018) found that Arabidopsis LBD16 interacted with bZIP59 protein to jointly initiate the expression of the downstream gene *FAD-BD*, thereby promoting callus formation. Their study found that the AtbZIP59-LBD complex is an auxin-induced callus. Key regulators of cell fate change during wound tissue formation, which may open the door to further exploration of the relationship between the remarkable regenerative capacity of plants and developmental plasticity. In this study, through yeast two-hybrid, BiFC, and Pull-down assays, we identified *IAA5* as an interacting protein of *ZmLBD2*. The Aux/IAA proteins are a large family of auxin coreceptors and transcriptional repressors, which are involved in auxin signaling. Aux/IAA proteins also play an important role in plant responses to abiotic stresses (Cheol Park et al., 2013; Wang F. et al., 2021). We found that the auxin content decreased under drought conditions, and the transcriptional expression levels of IAA-related genes were also affected. Jung et al. (2015) found that the rice Aux/IAA gene *OsIAA6* was highly induced by drought stress, thereby improving drought tolerance through the regulation of auxin biosynthesis genes. Therefore, we speculate that *ZmLBD2* interacts with *ZmIAA5* to regulate the expression of downstream auxin synthesis-related genes to improve the drought resistance of plants. We plan to further verify our hypothesis in future work.

## Conclusion

In conclusion, we show that the *ZmLBD2* gene is a positive regulatory factor for drought response. Subcellular localization results showed that the ZmLBD2 transcriptional protein was located in the nucleus. The *ZmLBD2* gene specifically binds with the inverted repeats of “GCGGCG”. Under drought stress, overexpression of ZmLBD2 improved drought resistance by increasing plant germination rate, root length, fresh weight, relative water content, chlorophyll, and proline content. In addition, it can also be overexpressed to promote the activity of CAT, SOD, and POD in *A. thaliana* to strengthen drought resistance by lowering ROS levels. Meanwhile, we also screened out the *ZmIAA5* gene interaction with *ZmLBD2*. This study provides a theoretical basis for the future analysis of the biological function of the interaction between maize LBD2 and IAA5 in auxin-regulated plant responses to drought stress.

## Data availability statement

The datasets presented in this study can be found in online repositories. The names of the repository/repositories and accession number(s) can be found in the article/**Supplementary Material**.

## Author contributions

SYG and YYM conceived research plans and designed experiments. PJ, XTW and ZZJ conducted experiments. PJ wrote the draft. SYL and ZZJ analyzed the data. PJ, SYG and YYM reviewed and edited this article and provided helpful comments and discussions. All authors contributed to the article and approved the submitted version.

## Funding

This work was supported by Jilin Province Science and Technology Development Plan Project [20220202008NC].

## Conflict of interest

The authors declare that the research was conducted in the absence of any commercial or financial relationships that could be construed as a potential conflict of interest.

## Publisher’s note

All claims expressed in this article are solely those of the authors and do not necessarily represent those of their affiliated organizations, or those of the publisher, the editors and the reviewers. Any product that may be evaluated in this article, or claim that may be made by its manufacturer, is not guaranteed or endorsed by the publisher.
