# Supplementary material for: ZmLBD2 a maize (Zea mays L.) lateral organ boundaries domain (LBD) transcription factor enhances drought tolerance in transgenic Arabidopsis thaliana
Source: Front Plant Sci. 2022 Oct 13;13:1000149. doi: 10.3389/fpls.2022.1000149 (PMC9612921; doi:10.3389/fpls.2022.1000149)
Supplement: Supplementary file 1 [file Table_1.doc]

**Table S1** List of primers used in this study

| **Name** | **Sequence 5’-3’** |
| --- | --- |
| ATACTIN8-qPCR-F | CTCAGGTATTGCAGACCGTATGAG |
| ATACTIN8-qPCR-R | CTGGACCTGCTTCATCATACTCTG |
| Zm18S-qPCR-F | CCTACGCTCTGTATACATTAGC |
| Zm18S-qPCR-R | GTGTTGAGTCAAATTAAGCCGC |
| ZmLBD2-qPCR-F | ATTGCACCTCCGGCCACC |
| ZmLBD2-qPCR-R | TTTGTCCAATTAATTAGGCTACGTA |
| AtCOR15A-qPCR-F | GGCCACAAAGAAAGCTTCAG |
| AtCOR15A-qPCR-R | CTTGTTTGCGGCTTCTTTTC |
| AtP5CS1-qPCR-F | GCGCATAGTTTCTGATGCAA |
| AtP5CS1-qPCR-R | TGCAACTTCGTGATCCTCTG |
| AtRD29A-qPCR-F | TGGACACGAATTCTCCATCA |
| AtRD29A-qPCR-R | TTCCAGCTCAGCTCCTGATT |
| AtNCED3-qPCR-F | ACCAACAAGAATGCCTTCCA |
| AtNCED3-qPCR-R | TAACAGAAACCAGCTGAGCTCGA |
| AtDREB2A-qPCR-F | GACCTAAATGGCGACGATGT |
| AtDREB2A-qPCR-R | TCGAGCTGAAACGGAGGTAT |
| AtABI4-qPCR-F | AATCCGATTCCACCACCGAC |
| AtABI4-qPCR-R | AGGGATACCGTACGGACCAA |
